# Nadir CA-125 serum levels during neoadjuvant chemotherapy and no residual tumor at interval debulking surgery predict prognosis in advanced stage ovarian cancer

**DOI:** 10.1186/s12957-020-01978-6

**Published:** 2020-08-13

**Authors:** Kazuto Nakamura, Yoshikazu Kitahara, Toshio Nishimura, Soichi Yamashita, Keiko Kigure, Ikuro Ito, Tatsuya Kanuma

**Affiliations:** 1 Department of Gynecology, Gunma Prefectural Cancer Center, 617-1, Takabayashinishi, Ota, Gunma, 373-8550 Japan; 2 Department of Obstetrics and Gynecology, Gunma University Hospital, Maebashi, Gunma 371-8511 Japan; 3 Department of Obstetrics and Gynecology, Takasaki General Medical Center, Takasaki, Gunma 370-0829 Japan

**Keywords:** Ovarian cancer, Neoadjuvant chemotherapy, Interval debulking surgery

## Abstract

**Background:**

Recent phase III randomized trials have suggested that neoadjuvant chemotherapy followed by interval debulking surgery (NACT-IDS) is a treatment option for patients with advanced epithelial ovarian cancer. This study aimed to use CA-125 and computed tomography (CT) scanning to generate a simple and clinically applicable model of predicting complete cytoreduction by interval debulking surgery (IDS) and the overall survival in patients who receive taxane/platinum-based chemotherapy as neoadjuvant chemotherapy (NACT).

**Methods:**

Patients with stage IIIc or IV epithelial ovarian cancer who underwent taxane/platinum-based NACT followed by IDS in Gunma Prefectural Cancer Center, Takasaki General Medical Center, and Gunma University from April 2009 to March 2015 were included. Patients underwent a CT scan to confirm confirm tumors unresectable by standard surgery before NACT. CA-125 levels were measured pre-NACT, after each cycle of NACT, and before IDS. CT was also performed before IDS to evaluate tumor metastasis. Data were collected retrospectively and analyzed to determine the predictive factors of complete resection and overall survival.

**Results:**

Among 63 patients who received NACT-IDS, 43 and 20 patients had stages IIIc and IV epithelial ovarian cancer at diagnosis, respectively. CT predictors of residual tumors after IDS such as extra-ovarian implants (*P* = 0.009) and omental cakes (*P* = 0.038) were not present. Univariate analysis revealed that the independent factors for overall survival were no residual tumor by IDS (*P* = 0.0016) and CA125 ≤ 20 U/ml before IDS (*P* = 0.0011).

**Conclusions:**

Although this study had a small sample size, NACT-IDS used to completely remove macroscopic disease which significantly improved the prognosis of patients with preoperative CA-125 ≤ 20 U/ml. Results from this study provide useful information for future studies on the management of patients with advanced epithelial ovarian cancer.

## Background

In Japan, 10,000 women are annually diagnosed with epithelial ovarian cancer (EOC). Although ovarian cancer is responsible for 28% of all malignant gynecologic tumors, it accounts for 42% of deaths from gynecologic malignancies. In advanced stage EOC, the standard treatment is primary debulking surgery (PDS) followed by platinum-based chemotherapy. Since two randomized controlled trials (RCT) [1, 2] have reported the non-inferior survival of neoadjuvant chemotherapy followed by interval debulking surgery (NACT-IDS) compared to PDS, the use of NACT-IDS for advanced EOC has increased. NACT-IDS has been used to achieve a higher rate of complete surgery and reduce surgical morbidity. Over the last decade, numerous retrospective studies and meta-analyses have found that patients with neoadjuvant chemotherapy (NACT) who had complete removal of all macroscopic tumors had an increased survival advantage [3–7]. However, the best method of evaluating which patients will benefit from NACT-IDS remains controversial.

In clinical practice, imaging studies such as computed tomography (CT), magnetic resonance imaging (MRI), and positron emission tomography (PET) scan are utilized to evaluate the extent of the disease. Among them, CT is the standard method for evaluating tumor spread in the pleural cavity, abdomen, and pelvis. However, the prediction accuracy for suboptimal cytoreduction of PDS varies between 71% and 93% [8, 9]. Even with newer methods such as spiral and multidetector CTs, the accuracy of CT in predicting complete cytoreduction is disappointing [10]. Diagnostic laparoscopy, another way to evaluate tumor distribution, has been demonstrated to be feasible and safe before PDS [11] and after NACT during interval debulking surgery (IDS) [12]. However, to date, no definitive method of imaging has been established to predict the absence of residual tumor by both PDS and IDS.

The measurement of CA-125 serum levels has been widely adopted as a biomarker for the management of patients with EOC. Several reports have shown correlations between CA-125 levels and resectability in PDS and IDS [13, 14]. However, CA-125 levels do not always reflect tumor burden. Based on the different criteria utilized by each study, the accuracy rate of CA-125 in predicting surgical outcomes for optimal cytoreduction is varied. Optimal cytoreduction has been defined by the Gynecologic Oncology Group as residual disease no greater than 1 cm in diameter [15]. Furthermore, several reports have indicated that patients whose tumors were debulked to no macroscopic disease had an increased survival advantage [16, 17]. Although optimal cytoreduction after NACT-IDS has been less analyzed compared to PDS [18], the goal of IDS according to the EORTC 55971 trial should be no gross residual tumor [1]. This study aimed to use CA-125 and CT scan to generate a simple and clinically applicable model of predicting complete cytoreduction by IDS and the patient’s overall survival.

## Methods

After receiving approval from the institutional review board of Gunma Prefectural Cancer Center (405-30081), we conducted a multicenter study. Three institutions belonging to the Gunma Local Medical Society were included: Gunma Prefectural Cancer Center, Gunma University, and Takasaki General Medical Center. The protocol of this study was also approved based on the guidelines set by the ethical committees of the two other institutions. Considering the retrospective nature of the study, informed consent was not obtained from each participant. Instead, all participants were given the right to withdraw their consent for use of their data.

The medical records of patients with International Federation of Gynecology and Obstetrics (FIGO) stages IIIc and IV tumors who received NACT-IDS between 2009 and 2015 were obtained from the aforementioned three institutions for analysis. Before undergoing NACT, all patients underwent a CT scan to confirm the presence of unresectable tumors by PDS, such as extensive peritoneal carcinomatosis, diaphragmatic confluent carcinomatosis, superficial liver metastasis, and/or mesenteric carcinomatosis. The decision to choose NACT-IDS was based on the attending physician’s judgment. NACT was continued until one of the following criteria was met: (1) CA-125 levels reached ≤ 20 U/ml, and (2) CA-125 levels reached a plateau. Demographic data, number of NACT cycles, and CA-125 levels pre-NACT, after each NACT cycle, and before IDS were obtained from the patients’ medical records. CT was also performed before IDS to evaluate tumor metastasis. All retrospectively collected data were analyzed to determine the predictive factors of complete resection and overall survival. All IDS were performed by gynecologic oncologists.

Table 1 summarizes the data on the patient’s age, tumor stage, tumor size before NACT, histology, progression-free survival, overall survival, pre-NACT CA-125, post-NACT CA-125, number of NACT cycles, and number of chemotherapy cycles after IDS. CT scan was used to determine the presence of extra-ovarian (peritoneal and mesentery) implants, omental metastasis, and ascites. Except for omental tumors, the maximal tumor sizes of extra-ovarian tumors before NACT were categorized into < 1 cm, 1–2 cm, 2–5 cm, and > 5 cm. After IDS, the group without residual tumor was defined as R0, while the group with residual tumor was defined as R1. 
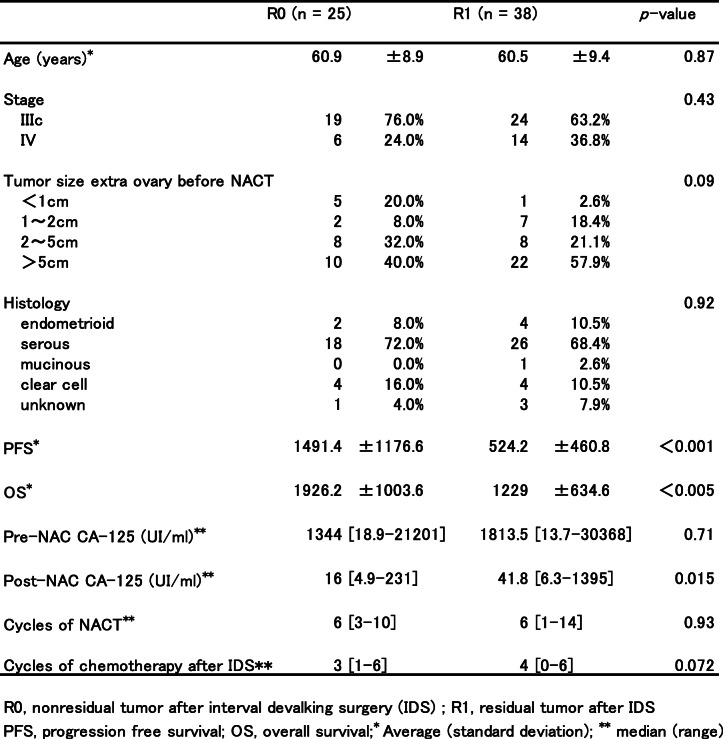

**Table 1:**
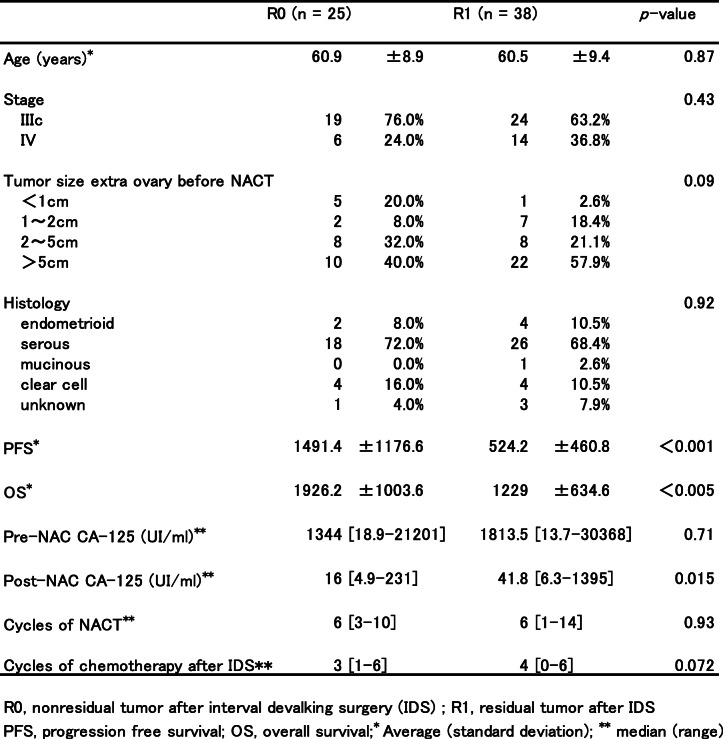
Patient characteristics

*T* test and Mann-Whitney *U* test were used to compare the differences in patients’ characteristics between the R0 and R1 groups. Chi-squared test was used to calculate the odds ratios and 95% confidence intervals (95% CI) for the presence or absence of residual tumor after IDS and for peritoneal dissemination, omental dissemination, and ascites (Table 2). The age-adjusted odds ratios for overall survival after IDS were calculated using logistic regression analysis (Table 3). Survival curves for overall survival and progression-free survival of the R0 and R1 groups were calculated using the Kaplan–Meier method and log-rank test. In addition, we subdivided the R0 and R1 groups into four groups depending on the CA-125 level. The survival curves for overall survival and progression-free survival of each group were then calculated using the Kaplan–Meier method. Furthermore, for each survival curve, the restricted mean survival time (RMST) was calculated with the *τ* value set to 2000 days. Across group, homogeneity was also tested. The *τ* value was set to 2000 days (approximately 5 years) because follow-up was to be performed for at least 5 years after IDS. 
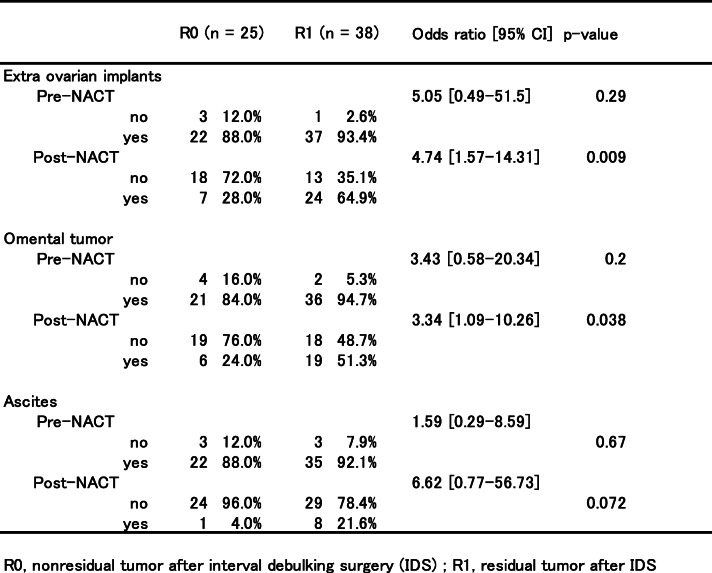

**Table 2:**
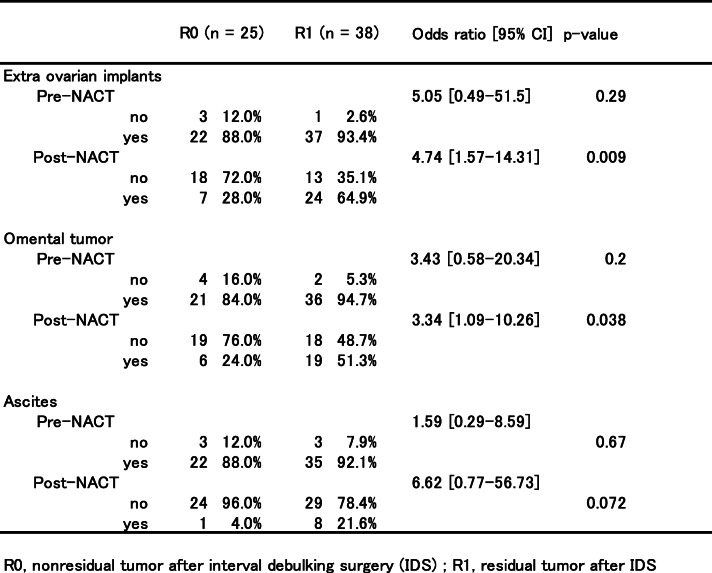
Univariate analysis of computed tomography prediction of no resudual tumor


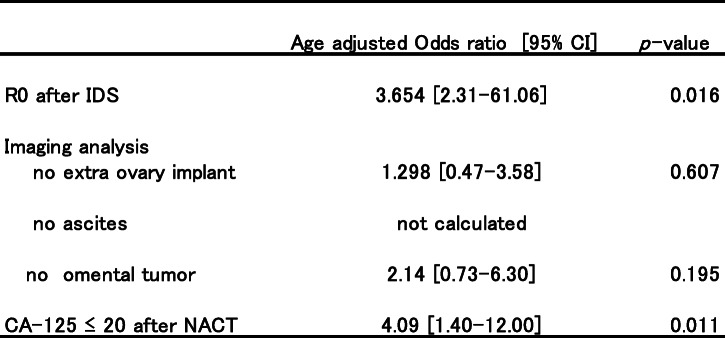

**Table 3:**
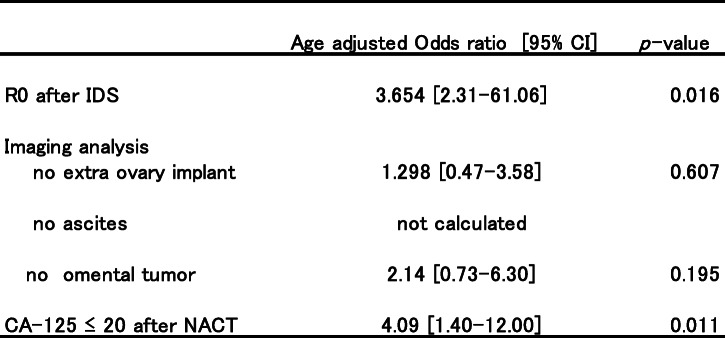
Univariate analysis for overall survival after interval debulking surgery

The *T*- and Mann-Whitney *U* tests were two-tailed. A *P*-value < 0.05 was considered statistically significant. All statistical analyses were performed using SAS ver. 9.4.

## Results

A total of 63 patients from three institutions under the Local Medical Society of Gunma Prefecture, Japan, were enrolled into this retrospective study. Table 1 summarizes the data on the patient’s age, tumor stage, tumor size before NACT, histology, progression-free survival, overall survival, pre-NACT CA-125, post-NACT CA-125, number of NACT cycles, and number of chemotherapy cycles after IDS. There were no significant differences among the R0 and R1 groups in terms of patient age (*P* = 0.87), tumor stage (*P* = 0.43), and tumor histology (*P* = 0.92) after IDS.

The largest tumor size before NACT outside the ovary was also not statistically different between groups (*P* = 0.09). However, the progression-free survival (*P* < 0.001) and overall survival (*P* < 0.005) were significantly longer in the R0 group. Furthermore, post-NACT CA-125 levels in the R0 group were significantly lower (*P* = 0.015) than those in the R1 group. Although the number of NACT cycles was not related to the residual tumor status after IDS (*P* = 0.93), one patient from both groups received more than 10 NACT cycles to meet the criteria described in the Methods section. Consolidation chemotherapy after IDS also showed no significant difference between the two groups (*P* = 0.072). We then investigated the radiological tumor response by comparing CT images at baseline and after NACT to predict no residual tumor by IDS. Table 2 lists the number and percentage of patients who underwent IDS for pre-NACT and preoperative CT findings. Pre-NACT values could not predict R0 after IDS, but extra-ovarian implants (*P* = 0.009) and omental tumors in post-NACT CT (*P* = 0.038) presented as statistically significant parameters for prediction of R0 in univariate analysis. By contrast, ascites after NACT between the two groups was not statistically significant (*P* = 0.072).

**Fig 1:**
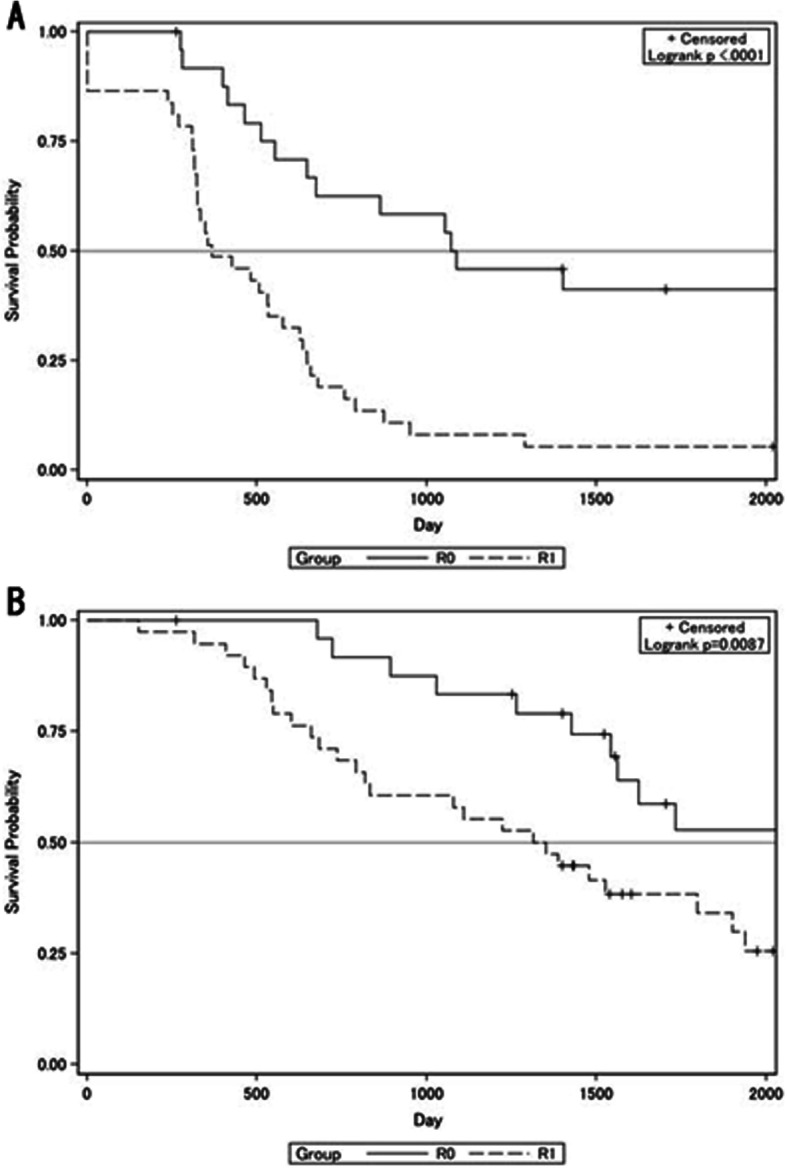
Survival by residual tumor status after interval debulking surgery. **a** Progression free survival and **b** overall survival in each residual tumor status (R0, no residual tumor. R1, residual tumor)

**Fig 2:**
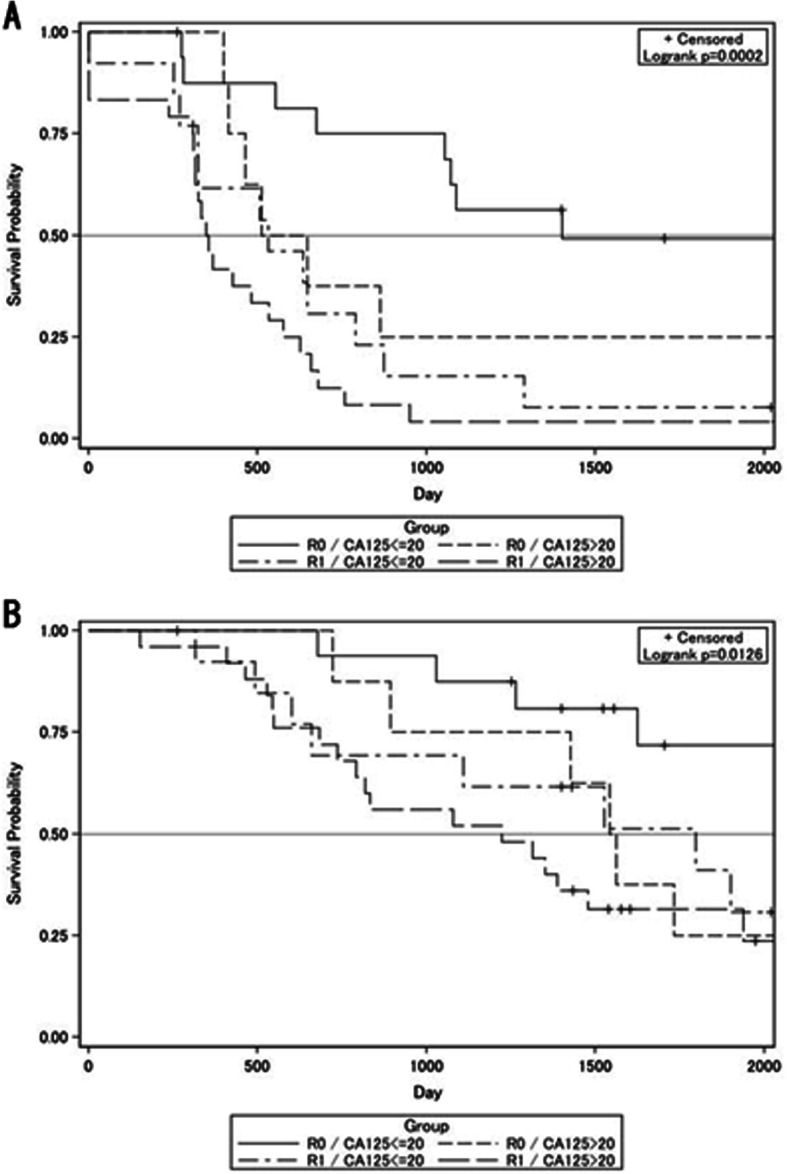
Survival by residual tumor status after ineterval debulking surgery and CA-125 level prior to IDS. **a** Progression free survival and **b** overall survival, stratified by residual tumor (R0, no residual tumor and R1, residual tumor) and CA-125 level (CA-125 ≤ 20 and CA-125 > 20)

We used RMST since some overall survival and progression-free survival curves did not reach a 50% survival probability. For R0 patients, the RMST of progression-free survival (Fig. 1a) was 1234.6 days (standard error [SE] ± 141.5), while the overall survival RMST (Fig. 1b) was 1664.3 days (SE ± 73.9). For R1 patients, the progression-free survival RMST (Fig. 1a) was 522.7 days (SE ± 73.9), while that for overall survival (Fig. 1b) was 1269.3 days (SE ± 102.5). Both progression-free survival (*P* < 0.0001) and overall survival (*P* = 0.039) were significantly longer in R0 patients. A stratified analysis of progression-free and overall survivals was carried out by splitting patients into four groups. Based on the residual tumor status and CA-125 levels, the RMST for progression-free survival was as follows: no residual tumor and CA-125 ≤ 20 U/mL, 1394.9 days (SE ± 165.0); no residual tumor and CA-125 > 20 U/mL, 912.1 days (SE ± 227.5); residual tumor and CA-125 ≤ 20 U/mL, 649.08 days (SE ± 139.2); and residual tumor and CA-125 > 20 U/mL, 454.2 days (SE ± 82.1) (Fig. 2a). The RMST for overall survival was as follows: no residual tumor and CA-125 ≤ 20 U/mL, 1773.3 days (SE ± 104.1); no residual tumor and CA-125 > 20 U/mL, 1484.6 days (SE ± 155.0); residual tumor and CA-125 ≤ 20 U/mL, 1395.4 days (SE ± 178.0); and residual tumor and CA-125 > 20 U/mL, 1205.9 days (SE ± 123.7) (Fig. 2b). In both progression-free (*P* < 0.0001) and overall survivals (*P* = 0.0049), the patient subgroup with no residual tumor and CA-125 ≤ 20 U/mL had better prognoses than the remaining three subgroups. We further analyzed factors associated with the overall survival of patients with R0 and R1 after NACT-IDS (Table 3).

R0 after IDS (odds ratio, 3.654 [95% CI, 2.31–61.06]) and CA-125 ≤ 20 U/mL after NACT (odds ratio, 4.09 [95% CI, 1.40–12.00]) were independent predictive factors for increased overall survival. However, while the absence of an omental tumor did not predict overall survival, it was shown to be associated with no residual tumor after IDS (Table 2). Since none of the patients who were alive had any detected ascites after NACT, we could not calculate the odds ratio of “no ascites.” In this study, one grade 1 and three grade 2 patients suffered from small intestine blockade. Additionally, three grade 3 patients required medical care for urinary tract problems. There was no treatment-related mortality.

## Discussion

Following the publication of two randomized studies [1, 2], NACT-IDS has become a standard option for the treatment of advanced EOC in many countries. However, consensus guidelines on optimal patient selection for NACT-IDS have not yet been established. Numerous studies have used CT and CA-125 levels to provide indicative parameters for patient selection of NACT-IDS. In this study, we analyzed data from 63 patients who underwent NACT-IDS and found that patients with no residual tumor after IDS and CA-125 ≤ 20 U/mL had a significantly longer overall survival.

The Society of Gynecologic Oncology and American Society of Clinical Oncology practice guidelines recommend that IDS should be performed after ≤ 4 cycles of NACT [19] since no RCTs have been conducted on IDS after > 4 NACT cycles. In support of the guidelines, retrospective studies have shown that patients who received extended NACT cycles had worse outcomes [20, 21]. However, other studies have reported contradictory results by demonstrating that IDS after ≥ 6 NACT cycles could be safe and help achieve higher complete resection [22–25]. The biological nature of a tumor acquiring chemotherapy resistance remains unclear. According to the Goldie–Coldman hypothesis [26], one could expect that extending the NACT treatment could raise concerns regarding the induction of indelible chemotherapy-resistant clones. On the contrary, treatment with ≤ 4 NACT cycles carries the risk of being insufficient. In our patient cohort, the median and average numbers of NACT cycles were 6 and 5.6, respectively. No severe adverse events were reported. Patients who achieved R0 after IDS had substantially improved overall survival (Fig 1b) Thus, we believe that instead of a fixed number of NACT cycles, patients should be offered a flexible number to reach R0 status. To confirm our hypothesis that alternating the timing of surgery is feasible, RCT should be conducted to focus on patient benefit, especially in prognostic outcomes over chemotherapy-related toxicity.

Several investigators have attempted to develop a model which directs patients to undergo either PDS followed by chemotherapy or NACT-IDS. Patients’ responses to NACT are usually monitored by imaging analysis such as CT, MRI, and PET scans at adequate intervals and by measurements of CA-125 levels after each course of chemotherapy. A number of studies have developed models using CT to predict optimal debulking at PDS by setting precise parameters for evaluation [8, 9, 27, 28]. However, each study utilized a different set of parameters. This has resulted in variable R0 predictions. Ghisoni et al. proposed a predictive model for optimal cytoreduction at IDS by utilizing parameters such as age and CA-125 levels at diagnosis. The peritoneal cancer index was assessed during laparoscopy at IDS [29]. Among those parameters, the peritoneal cancer index was the most weighed since it had the most positive predictive value of incomplete cytoreduction at IDS. In this study, we adopted a simple model for predicting surgical outcomes after IDS. It may be easily applied to clinical practice and help prevent multicenter study bias (Table 2). Our model shows that both extra-ovarian implants and omental tumor after NACT are predictive features for R0. This result is partially supported by the fact that the omentum is an important site for the assessment of chemotherapy response [30]. A few studies have predicted the surgical outcome prior to IDS [31, 32] by describing the radiological evaluation prior to IDS. This was associated with chemotherapy response but not progression-free or overall survival. Consistently, the absence of residual tumors in patients receiving NACT-IDS is prognostically less pronounced than that in patients who undergo PDS [33, 34]. Therefore, one could expect that non-visible residual tumors might remain after NACT-IDS.

Previous studies have investigated whether the serum CA-125 level prior to IDS is a predictive factor of surgical outcome [14, 28, 35]. The Gynecologic Cancer Intergroup CA-125 disease progression criteria uses 35 U/ml as the normal upper limit [36]. In general, the 35 U/mL limit may be appropriate. However, this cut-off may be falsely negative for interpreting the CA-125 level of the patients in this study who were either postmenopausal or had compromised ovarian functions under chemotherapy. Since it has been well accepted that the CA-125 level is affected by estrogen and menopausal status, some studies have demonstrated that the upper limit of the normal CA-125 level is no more than 20 U/ml [37, 38]. Despite including patients treated by PDS and maintenance chemotherapy, the Southwest Oncology Group and Gynecologic Oncology Group clinical trial observed differences in the median progression-free survival of patients after PDS between CA-125 levels ≤ 10 U/ml, 11–20 U/ml, and 21–35 U/ml [39]. In this study, we did not find statistical differences in progression-free and overall survivals between CA ≤ 10 U/ml and 11–20 U/ml (data not shown). Collectively, we set 20 U/ml cutoff for this study. Hence, we attempted to draw Kaplan–Meier survival curves for progression-free and overall survivals based on the residual tumor status at IDS and CA-125 levels ≤ 20 U/ml prior to IDS (Fig. 2). Since the univariate analysis showed that both R0 at IDS and CA-125 ≤ 20 U/ml after NACT were critical for a better prognosis (Table 3), we believe that it is insufficient to merely target R0 by IDS. Moreover, we need to develop new biomarkers which could precisely detect remaining minimal tumors and aid in strategizing the management of patients with advanced EOS.

In summary, we demonstrated that R0 at IDS and CA-125 ≤ 20 U/mL after NACT are favorable factors for overall survival in advanced EOC. However, this study has some limitations, including its small sample size and retrospective nature. The results of our study need further validation by future studies and RCTs with larger cohorts. Furthermore, it is important to note that this study was conducted in patients who were treated during the era of taxane–platinum therapy. Thus, consolidation and maintenance therapy after NACT-IDS may be altered with emerging drugs such as poly (ADP-ribose) polymerase inhibitors and immune checkpoint inhibitors.

## Data Availability

The data are not available due to institutional policy.

## Abbreviations

NACT: Neoadjuvant chemotherapy
IDS: Interval debulking surgery
CT: Computed tomography
EOC: Epithelial ovarian cancer
PDS: Primary debulking surgery
RCT: Randomized controlled trial
MRI: Magnetic resonance imaging
PET: Positron emission tomography
RMST: Restricted mean survival time
